# Diet and Oral Squamous Cell Carcinoma: A Scoping Review

**DOI:** 10.3390/ijerph21091199

**Published:** 2024-09-10

**Authors:** Marcela Gomes Reis, Lucas Carvalho Lopes, Ana Beatriz Amaral M. De A. Sanches, Nathalia Sernizon Guimarães, Roberta Rayra Martins-Chaves

**Affiliations:** 1 Faculty of Medical Sciences of Minas Gerais, Alameda Ezequiel Dias, 275, Belo Horizonte 30130-110, MG, Brazil; reis.marcelanutri@gmail.com (M.G.R.); lucas_lopes@cienciasmedicasmg.edu.br (L.C.L.); ana_sanches@cienciasmedicasmg.edu.br (A.B.A.M.D.A.S.); 2 Observatory of Epidemiology, Nutrition and Health Research (OPENS), Alameda Ezequiel Dias, 275, Belo Horizonte 30130-110, MG, Brazil; 3 Department of Nutrition, School of Nursing, Federal University of Minas Gerais, Alfredo Balena Avenue, 190, Room 314, Santa Efigênia, Belo Horizonte 30130-100, MG, Brazil; 4 Center for Molecular Studies in Oncology (NEMO), Alameda Ezequiel Dias, 275, Belo Horizonte 30130-110, MG, Brazil

**Keywords:** diet, squamous cell carcinoma of head and neck, mouth neoplasms, public health

## Abstract

Oral squamous cell carcinoma (OSCC) is the sixth most common type of cancer globally. While smoking is a key risk factor, rising cases in non-smokers highlight the need to explore other factors like diet. This scoping review aims to deepen the evidence on the relationship between OSCC and diet, following PRISMA-ScR guidelines, and was registered on Open Science Framework. Searches were performed in four electronic databases: MEDLINE, Embase, Web of Science, and Lilacs, without date or language restrictions. Studies were evaluated, extracted, and compiled in a narrative table. Seventeen studies with 10,954 patients were analyzed. Most patients were male (74.63%), aged 18–89 (average 50.62). Studies were mainly from high (82%) and medium (17%) Human Development Index (HDI) countries. Dietary surveys included a Food Frequency Questionnaire (FFQ) (58.8%), interviews/questionnaires (17.6%), and an FFQ with a photographic atlas (5.9%). Certain foods in excess like fruits, vegetables, and tea were inversely associated with OSCC, while salty meats, dairy, coffee, sausages, and fried and spicy foods were positively associated. Due to the heterogeneity of the tools used to obtain food frequency data, the results should be interpreted cautiously. New standardized studies and randomized trials are essential to advance understanding and control confounding factors in this field.

## 1. Introduction

Oral squamous cell carcinoma (OSCC) is the most prevalent head and neck cancer, with approximately 350,000 new cases each year worldwide [1,2]. Alcohol and tobacco cause irreversible DNA damage in the squamous cells lining the oral mucosa [3,4], representing the main etiological factors for OSCC initiation [5]. Other environmental risk factors such as oral microbiome imbalance [6,7,8], chronic mechanical trauma [9], and dental implants have also been studied in the OSCC context, but their etiopathogenic role is still unclear [10,11]. Recent studies have associated eating patterns with several malignant neoplasms’ oncogenesis [9,10] and show that a healthy diet, rich in fresh fruits and vegetables and bioactive compounds, offers a synergy of antioxidant, anti-inflammatory, anti-angiogenic, and anti-proliferative properties [12,13,14]. These properties can potentially control cancer cells, preventing or treating the disease [13].

For OSCC, the high heterogeneity among studies assessing food consumption prevents a strong substantial association between eating patterns and tumor development. Therefore, synthesizing the literature regarding eating patterns in OSCC patients is vital to characterize the key most consumed food in this group of patients. Providing a broad perspective on this subject may aid the inclusion of preventive nutritional advice in the existing OSCC prevention recommendations [15]. None of the previous literature has encompassed such a wide range of foods and food groups for this type of cancer in a single study. In this scoping review, we evaluated the published literature on the eating patterns of OSCC patients and performed a detailed descriptive analysis of their nutritional landscape.

## 2. Materials and Methods

This study aimed to deepen the evidence on the relationship between OSCC and diet through a scoping review, mapping the existing literature on the relationship between diet and OSCC and conducting a detailed descriptive analysis of the findings to identify research gaps on this topic. It was developed according to the Joanna Briggs Institute tool (JBI) [16] and reported according to Preferred Reporting Items for Systematic Reviews and Meta-Analyses extension for Scoping Reviews (PRISMA-ScR) [17]. This study was registered on the Open Science Framework (https://osf.io/f4ndm/, accessed on 1 March of 2024) [18].

### 2.1. Data Source and Search Strategy

The searches were performed in four electronic databases MEDLINE, Embase, Web of Science, and Lilacs, with no date or language restrictions. The studies were evaluated for eligibility, extracted, and compiled in a narrative form. Additionally, we hand-searched the reference lists of the included studies. The last search was conducted in March 2024.

Descriptors were identified in Medical Subject Headings (MeSH), Descritores em Ciências da Saúde (DeCS), and Embase Subject Headings (Emtree). These were combined with the Boolean operator AND, while their synonyms were combined with the Boolean operator OR. The following MeSH terms formed the search strategy used, which was adapted based on descriptors in each database, presented in Table S1.

The acronym PCC (P—population; C—concept; C—context) was used to formulate the central question. The guiding question was “What are the different associations of this type of cancer with diet?” (Table 1). The research strategy was developed with the support of a collaborator experienced in review studies (N.S.G.).

### 2.2. Eligibility Criteria

We included quantitative and qualitative observational studies that evaluated individuals aged ≥ 18 years with OSCC and used questionnaires that carried out a descriptive assessment of dietary patterns, whether validated or not. Reviews, preclinical studies, interventional studies, ecological studies, cost-effectiveness analyses, letters, and editorials were excluded. Studies that evaluated pregnant or lactating women, children, or adolescents and studies involving individuals who used chronic supplementation or carcinogenic herbs (areca nut derivate and betel quid), alcohol, or tobacco were excluded.

### 2.3. Study Selection and Data Extraction

The studies found in the electronic search of the databases were exported in “ris” format to the Rayyan Qatar Computing Research Institute application for systematic reviews [19]. The title and abstracts were independently screened by two reviewers (LCL and ABAMAS) to determine whether they met the inclusion criteria. After this stage, the textual analysis of the studies was carried out. Three independent reviewers (NSG, RRMC, and MGR) analyzed any discrepancies. To create the extraction table in an Excel^®^ 2013 spreadsheet, the following data were collected: reference (author, year, and title), study location, study design, follow-up period (weeks), age, gender, lesion location, foods analyzed (type or group), eating patterns evaluated, and main results for the outcomes assessed. In addition, we used it to calculate the mean and standard deviation (SD).

### 2.4. Data Description

Different studies employ varying methods to categorize the same food groups. Therefore, to understand the relationship between the pattern of consumption of each group and OSCC compared to the control group, we analyzed individual consumption within each study and frequency of consumption. This approach enabled us to determine the frequency of consumption for each food analyzed in both the OSCC and the control group without OSCC. Then, we calculated average frequencies to discern the eating patterns by food group between patients with OSCC and the control group. Finally, we conducted individual assessments of the eating patterns of OSCC patients to identify the most and least frequently consumed food types.

Fruit consumption was categorized into various subgroups, including fruit, fresh fruit, citrus fruits, oranges, apples, bananas, tomatoes, and all fruit. Leafy vegetables were categorized as green vegetables, yellow vegetables, cruciferous vegetables, lettuce, group A greens (green leaves), group B greens (others), and group C greens (roots and tubers). Red meat, chicken, and fish were categorized into red meat, chicken, fish, fresh meat, salted meat, and barbecue. Dairy and dairy products were categorized into dairy products and milk. Cold cuts were categorized as bacon and sausages. The results regarding the frequency of culinary preparations revealed that most OSCC patients consume fried and spicy/peppery foods, and hot or very hot beverages. The beverages assessed included infusions and teas categorized into various types, including general tea, coffee, mate tea, green tea, and black tea.

## 3. Results

**Figure 1:**
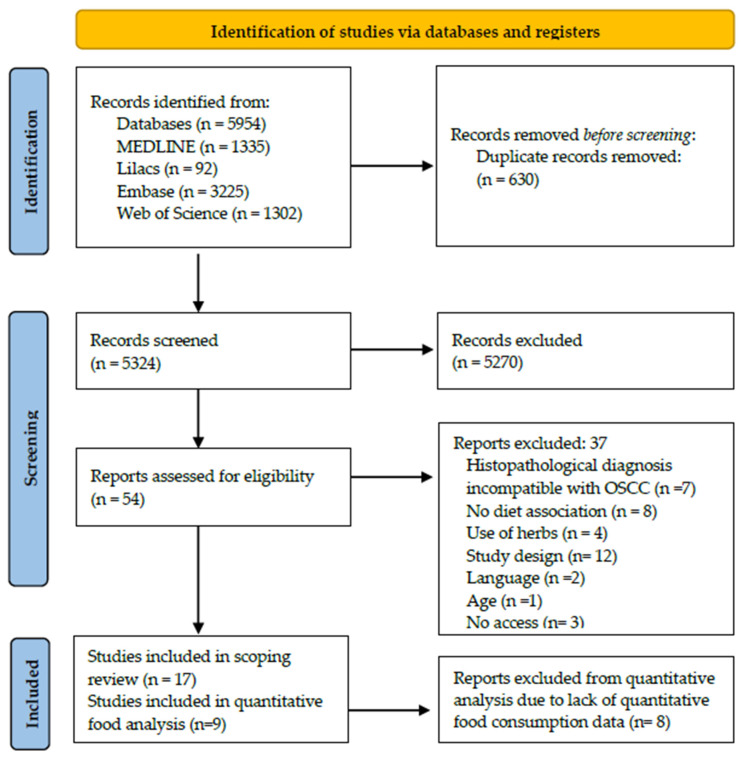
Flowchart for the selection of studies, 2024.

Our initial search retrieved 5954 studies in MEDLINE (via PubMed), Embase, Web of Science, and Lilacs. After excluding 630 duplicates, 5324 titles and abstracts were screened. Full-text articles for the remaining 54 records were retrieved, of which 37 were excluded according to the eligibility criteria described in Table 2 and Table 3. No records were included through hand-searching. Thus, 17 studies were included in the scoping review, 8 of which were excluded from the quantitative consumption analysis due to a lack of data (Figure 1).

### 3.1. Study Characterization

Regarding design, 14/17 papers were case–control studies (82%), two were cross-sectional studies (11%), and one was a cohort study (5%). The studies were conducted in South America (*n* = 7 studies; 41%), Asia (*n* = 6 studies; 35%), North America, and Europe (with 2 studies each; 11%). Regarding the Human Development Index (HDI), only high (*n* = 14 studies; 82%) and medium (*n* = 3 studies; 17%) HDI countries were analyzed, with the highest-ranking country being the United Kingdom (15th position) and the lowest being India (134th position).

### 3.2. Sample Description

The seventeen included studies described a total of 10,954 patients, of whom 8175 (74.63%) were male and 2059 (18.79%) female. Two studies did not identify the sex of their participants. The age ranged from 18 to 89 years (50.62 ± 3.0). Tumor location was only reported by five studies. The tongue was the most common anatomic location (*n* = 182 patients; 4.43%; $\bar{x}$ 36.40 ± 31.0), followed by floor of the mouth (*n* = 27 patients; 0.65%; $\bar{x}$ 6.75 ± 4.7) and the hard palate (*n* = 8 patients; 0.19%; $\bar{x}$ 2.67 ± 1.15).

The average follow-up time was 221.44 ± 152.12 weeks, as described in 16 studies. We categorized the studies into three groups, studies with a high follow-up time (≥105 weeks), studies with an intermediate follow-up time (53–104 weeks), and studies with a low follow-up time (≤52 weeks). Eleven studies (68.8%) presented a high follow-up time, two studies (12.5%) had an intermediate follow-up, and three (18.7%) studies had a low period of follow-up.

Schooling was categorized according to the length of study in years, with the highest proportion of study participants having completed between 0 and 5 years (*n* = 4342 individuals; 65.49%), considered to be low schooling, followed by medium schooling, with 6 to 12 years of study (*n* = 1283 individuals; 19.35%) and, finally, completion of more than 13 years (*n* = 1005 individuals; 15.15%), equivalent to high schooling. Other socio-demographic information such as income, social vulnerability index, occupation, and marital status, were not described in the studies evaluated.

### 3.3. Quantitative and Qualitative Eating Patterns Description

Regarding the type of dietary survey, ten studies (58.8%) used only the Food Frequency Questionnaire (FFQ), three studies used interviews and pre-structured questionnaires, another three studies used both questionnaires and self-reporting (17.6%, respectively), and only one study (5.9%) approached patients with the FFQ plus a photographic atlas for further instruction.

From each food group analyzed, we obtained a categorization of the overall average and specifically for each type of food, depending on the frequency of food consumption. This makes a description of each of the groups necessary. The general characteristics of the studies analyzed and the individual frequency of food consumption for the OSCC group are described in Table 2 and Table 3, respectively.

### 3.4. Fruits and Vegetables

Consumption of fruit three or more times a week was correlated with a lower frequency of OSCC. Conversely, the absence of fresh fruit consumption was correlated with a higher frequency of OSCC. In the vegetable food group, only carrots were evaluated. A minority of OSCC patients reported carrot consumption of two or more times a day (33.33%), whereas the majority reported consuming carrots less than once a day (56.20%).

### 3.5. Leafy Vegetables

The frequency of consumption of all these leafy vegetables was low for the majority of OSCC patients, who reported consuming them less than once or three times a week.

### 3.6. Red Meat, Chicken, and Fish

Interestingly, 36% of OSCC participants reported never consuming red meat, while 58% ate red meat one or more times a week. Accordingly, consumption of salted meat once a week or more was reported by 75% of participants, while 46% never consumed it. In general, the habit of never consuming red meat, or consuming it less than once a day, week, or month, was less frequently reported by OSCC individuals. Therefore, in the population of individuals with OSCC, a high frequency of red meat consumption was observed, with the sole exception of chicken, for which lack of consumption was predominant.

### 3.7. Dairy Products

Among OSCC patients, 45% reported consuming milk two or more times a day, while 54.5% consumed it less than once a day. Moreover, only 25% of participants indicated never consuming dairy products, while 47% consumed them one or more times a week.

### 3.8. Cold Cuts

Approximately 70% of patients with OSCC consume bacon two or more times a day, whereas 30% abstain from this food entirely. Conversely, the frequency of sausage consumption was more evenly distributed, with 46% of patients reporting no consumption and 39% consuming it two or more times a month.

### 3.9. Culinary Preparations and Beverages

Notably, 80.7% of the surveyed OSCC patients reported eating fried foods two or more times a week, while 30.8% indicated that they never consume it. Furthermore, 49% of participants with OSCC reported consuming spicy/peppery foods one or more times a week, and 33% stated that they consume it less than once a week. Finally, 52% of OSCC patients consume very hot beverages three or more times a week, and 31% consume hot foods two or more times a day.

Mate tea was the most frequently consumed beverage among OSCC participants, with 55.6% reporting that they drank it once a day or more, in contrast to 36% who did not consume it. Green and black tea frequency was evenly distributed. Finally, nearly 44% of OSCC participants stated consuming coffee three or more (29.9%) or two or more (14.3%) times a day.

## 4. Discussion

The World Cancer Research Fund International and the American Institute for Cancer Research state that eating fruits and vegetables is associated with a lower risk of various types of cancer, including oral cancer [20,21]. This can be explained by the properties of several bioactive compounds found in these foods, such as lycopene, resveratrol, flavonoids, isothiocyanates, and other minerals like magnesium and folate. These compounds have antitumor, antioxidant, and antiproliferative properties that enhance the immune system. They can also influence cellular mechanisms related to cell cycle regulation, DNA repair, and reactive oxygen species elimination [14].

Our research findings synthesize and illustrate the eating patterns adopted by OSCC patients. It was observed that fruits and vegetables were less frequently consumed than red meat, dairy products, cold cuts, fried or spicy foods, and hot or very hot beverages among OSCC patients.

The increased consumption of meat, especially salted and red meat, may lead to excessive iron storage or oxidative stress resulting from the free radicals related to red meat digestion [20,22]. In addition to the digestive metabolic alterations, red meat carcinogens can also be generated or increased according to the preparation/preservation method adopted. Interestingly, our results revealed a high frequency of cold cut, particularly bacon, and fried food consumption among OSCC patients. The relationship between the frequency of cancer in general and dairy products is still not well understood in the literature. However, it is known that dairy products are rich in calcium, vitamin D, and conjugated linoleic acid, which can influence cell specificity and differentiation, providing anti-cancer effects [23]. On the other hand, their high levels of fats and potential contaminants can make them pro-carcinogenic [24]. Therefore, it is important to consider that, like most foods, excessive consumption of dairy products does not provide benefits, while inadequate consumption fails to capture their important and nutritious properties [23]. While the frequency of milk consumption was evenly distributed among the OSCC patients included in our review, 47% consume other dairy products one or more times a week, and 25% indicated never consuming dairy products.

Daily coffee consumption may be beneficial due to its variety of biologically active compounds, known as antioxidants, and its ability to modulate apoptotic response and reverse cell cycle checkpoint function [25]. However, despite some studies, such as those cited in the systematic review by Li et al., 2016 [26], suggesting that high coffee consumption reduces the risk of oral cancer, it is noteworthy that any excesses are not safe. In our review, nearly 44% of OSCC participants stated consuming coffee three or more (29.9%) or two or more (14.3%) times a day. Similarly, with coffee, the literature supports the protective effect of tea against OSCC through the action of its antioxidant compounds, such as polyphenols and catechins, which act as scavengers of reactive oxygen species and can influence transcription factors and enzymatic activities [27]. We retrieved the OSCC patients’ consumption of green, black, and mate tea. Although green and black tea were evenly consumed by this population, 55.6% of the sample reported a daily consumption of one or more portions of mate tea, while 36% stated that they did not consume it [27].

The higher consumption of spicy and hot foods, as well as very hot temperatures, has been linked to a greater frequency of OSCC [28,29]. These subgroups should be considered based on their ability to cause mechanical and chemical sensitivity of the local mucosa. An interesting finding of our work was that 80.7% of the OSCC patients surveyed reported consuming fried food at least twice a week, while 30.8% stated they never eat fried food. We also found that 52% of OSCC patients consume very hot beverages three or more times a week, and 31% consume hot beverages two or more times a day. A controversial finding is that capsaicin, a compound found in peppers and spicy foods, may act as a cancer suppressant through its antioxidant and anti-inflammatory effects; in addition, it is one of the groups of crops that receives the largest amount of pesticides. Given its benefits, consuming spicy foods may lead to a lower risk of OSCC [30]. However, paradoxically, the low consumption of spicy food by the OSCC population may be associated with the symptoms of the disease [28,29]. In our review, 49% of the OSCC participants reported eating spicy or peppery food at least once a week, while 33% mentioned consuming it less than once a week.

### Validity, Limitations, and Prospects

The main limitation of this scoping review is the high heterogeneity among the included studies. To mitigate this, we categorized the results based on the type of foods and frequency of consumption for a descriptive analysis. While this approach enabled us to overcome this limitation for the current data, it is imperative to interpret our results with caution. We strongly advocate for further efforts to develop a standardized nutritional assessment tool for future observational studies that will help in elucidating the relationship between diet and OSCC risk.

The assessment of eating patterns represents a challenging task due to several errors in the application and interpretation of the different dietary survey protocols. Despite the absence of a gold-standard protocol, the Food Frequency Questionnaire (FFQ) is a low-cost and minimally participant-dependent survey method. Therefore, this approach has been used in various epidemiological and dietary investigative studies. Some other surveys include 24 h dietary recall (R24H) and self-reporting. However, these methods tend to omit foods/groups and rely solely on memory bias, being affected by patients’ memory issues due to aging or misunderstandings caused by educational limitations [31,32]. Since old age and low levels of schooling were predominant among our included population, this might affect the reliability of our results.

**Figure 2:**
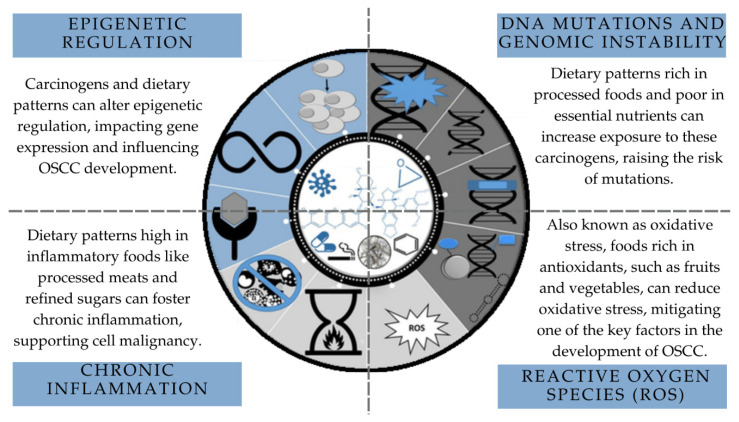
Mechanistic view of cancer modulation and associations with dietary patterns. Source: adapted from Guyton et al., 2018, Chemical Research in Toxicology [33].

Investigating whether a specific dietary pattern can cause and sustain molecular alterations until the development of OSCC is challenging due to the complexity of molecular events associated with oral carcinogenesis (Figure 2) [33]. To understand how diet influences the genetic and epigenetic mechanisms associated with oral cancer, it would be necessary to select a sample of individuals whose confounding variables could be controlled so that molecular assays could be carried out. Furthermore, the standardization of the methods used to collect data on dietary patterns is fundamental for a more consistent characterization of the dietary profile of individuals affected by OSCC.

In addition, the lack of data in the studies may have introduced some confounding factors, such as not describing whether the vegetables are canned or fresh, whether they are organic or grown with pesticides, and how they are prepared (for example, if they are fried). There was also a lack of control for risk factors already well established in the literature that can influence diet, such as the use of areca nut derivatives, chronic smoking, and alcohol consumption.

## 5. Conclusions

Our results comprehensively synthesize the nutritional landscape of OSCC. We found a high frequency of red meat and cold cut consumption and a low frequency of fruit, vegetable, and leafy vegetable consumption among OSCC patients. Although this eating pattern has been described as oncogenic for other malignant neoplasms, oral carcinogenesis is a multifactorial process. Therefore, further studies are needed to confirm the carcinogenic potential of dietary habits for OSCC initiation and development. It is also noteworthy that most foods are influenced by the quantity of consumption and not just by their quality. However, due to the heterogeneity of the tools used to obtain food frequency data, the results should be interpreted with caution. Standardized studies are essential to advance understanding in this field.

The development of preventive randomized clinical trials or prospective cohort studies on this subject is crucial to control for confounding factors related to this hypothesis. In addition, it is essential to understand and control risk factors and confounding variables, adjusting them appropriately for this specific population, given the lack of robust data that provide reliable and clear information to the scientific community and professionals, from prevention to diagnosis.

## Figures and Tables

**Table 1:** PCC acronym and question components.

Abbreviation	Description	Question Components
P	Population	Patients diagnosed with OSCC (experimental group) and without OSCC (control group)
C	Concept	Association between food and eating patterns and the disease
C	Context	Food and eating patterns

**Table 2:** General characteristics of the studies analyzed.

Food Group	Participants in Total (*n*)	Participants with OSCC (*n*)	Studies Analyzed	Mean Frequency of OSCC (%)	Mean Frequency of Control Group (%)
Fruits	5227	2256	5	46.88	53.13
Vegetables	592	296	1	52.06	47.94
Leafy Vegetables	4132	1616	5	47.83	52.17
Red Meat, Chicken, and Fish	2567	1198	2	48.66	51.34
Dairy Products	924	433	2	45.98	54.02
Infusions	1701	3698	4	30.30	69.70
Cold Cuts	1061	542	2	53.30	46.61
Fried Preparations	427	296	1	50.84	49.16
Spicy Preparations	592	187	1	41.34	41.34
Beverages—Temperature	427	187	1	41.79	58.21

Legend: OSCC—oral squamous cell carcinoma.

**Table 3:** Qualitative and quantitative description of food and beverage consumption frequencies in OSCC patients.

Food Group	Lowest Frequency Reports of Food Consumption in OSCC Patients			Highest Frequency Reports of Food Consumption in OSCC Patients		
	Type of Food	Quantitative Consumption	Frequency (%)	Type of Food	Quantitative Consumption	Frequency (%)
Fruits	Fruits	Three or more times a week	13.74%	Fruits	Less than three times a week	34.46%
	Fresh Fruit	One or more times a week	20.00%	Fresh Fruit	Never	46.88%
	Citrus Fruits	One or more times a week	30.15%	Citrus Fruits	Less than once a week	55.70%
	Orange	Two or more times a day	30.77%	Orange	Less than once a week	57.58%
	Apple	Two or more times a day	35.09%	Apple	Less than once a week	57.52%
	Banana	Two or more times a day	36.25%	Banana	Less than once a week	58.51%
	All fruits	Two or more times a day	37.58%	All fruits	Less than once a week	61.15%
	Tomato	Two or more times a day	41.52%	Tomato	Less than once a week	100.00%
Vegetables	Carrot	Two or more times a day	33.33%	Carrot	Less than once a day	56.20%
Leafy Vegetables	Green Vegetables	Once a day or more	18.94%	Green Vegetables	Less than once a week	56.70%
	Yellow Vegetables	One or more times a week	35.86%	Yellow Vegetables	Less than once a week	63.41%
	Cruciferous Vegetables	One or more times a week	37.01%	Cruciferous Vegetables	Less than once a week	56.85%
	Vegetable Group C (Roots and Tubers)	Three or more times a week	37.50%	Vegetable Group C (Roots and Tubers)	Less than three times a week	54.87%
	Lettuce	Two or more times a day	41.41%	Lettuce	Less than once a week	63.75%
	Vegetable Group A (Green Leaves)	Three or more times a week	45.71%	Group A Vegetables (Green Leaves)	Less than three times a week	58.33%
	Vegetables	One or more times a week	45.76%	Vegetables	Never	62.50%
	Vegetable Group B (Others)	Never	50.00%	Vegetable Group B (Other)	One or more times a day	50.89%
Red Meat, Poultry, and Fish	Red Meat	Never	35.97%	Red Meat	One or more times a week	58.22%
	Chicken	Less than once a week	37.06%	Chicken	Never	55.77%
	Fish	Less than once a week	38.22%	Fish	One or more times a week	52.38%
	Fresh Meat	Less than once a day	41.36%	Fresh Meat	Once a day or more	53.14%
	Salted Meat	Never	46.05%	Salted Meat	Once a week or more	75.00%
	Barbecue	Less than once a month	47.33%	Barbecue	Three or more times a month	50.00%
Dairy Products	Dairy Products	Never	25.00%	Dairy Products	One or more times a week	47.37%
	Milk	Two or more times a day	45.04%	Milk	Less than once a day	54.50%
Cold Cuts	Bacon	Never	30.19%	Bacon	Two or more times a day	68.32%
	Sausages	Never	46.67%	Sausages	Two or more times a month	38.71%
Fried Preparations	Fried Food	Never	30.87%	Fried Food	Two or more times a week	80.77%
Spicy Preparations	Spicy and Peppery Foods	Less than once a week	33.56%	Spicy and Peppery Foods	One or more times a week	49.11%
Beverages—Temperature	Hot	Two or more times a day	31.21%	Very Hot	Three or more times a week	52.36%
Infusions and teas	Coffee	Two or more times a day	14.35%	Coffee	Three or more times a day	29.92%
	Mate Tea	Never	36.00%	Mate Tea	Once a day or more	55.63%
	Green Tea	Two or more times a day	42.98%	Green Tea	Less than once a day	50.60%
	Black Tea	One or more times a day	44.87%	Black Tea	Less than once a day	46.69%

## Data Availability

This study used data available on public websites and electronic data banks. The Brazilian government gained access to the Embase platform (via the CAPES website).

## Supplementary Materials

The following supporting information can be downloaded at: https://www.mdpi.com/article/10.3390/ijerph21091199/s1, Table S1: Indexers used to select publications; Table S2: Full-text excluded articles and reasons. References [34,35,36,37,38,39,40,41,42,43,44,45,46,47,48,49,50,51,52,53,54,55,56,57,58,59,60,61,62,63,64,65,66,67,68,69,70,71,72,73,74,75,76] are cited in the Supplementary Materials.

## Abbreviations

OSCC	Oral squamous cell carcinoma
HDI	Human Development Index
FFQ	Food Frequency Questionnaire
PRISMA-ScR	Preferred Reporting Items for Systematic reviews and Meta-Analyses extension for Scoping Reviews
JBI	Joanna Briggs Institute
MeSH	Medical Subject Headings
DeCS	Descritores em Ciências da Saúde
Emtree	Embase Subject Headings
CAPES	24 h dietary recall (R24H); Coordenação de Aperfeiçoamento de Pessoal de Nível Superior
PDPG	Postgraduate Development Program
