# Supplementary material for: Diet and Oral Squamous Cell Carcinoma: A Scoping Review
Source: Int J Environ Res Public Health. 2024 Sep 10;21(9):1199. doi: 10.3390/ijerph21091199 (PMC11431716; doi:10.3390/ijerph21091199)
Supplement: Supplementary file 1 [file ijerph-21-01199-s001.zip › ijerph-3169270-supplementary.pdf]

Supplementary Materials

Table S1. Indexers used to select publications.

PubMed<br>1335	(“carcinoma, squamous cell”[MeSH Terms] OR “carcinomas squamous cell”[All Fields] OR “Squamous Cell Carcinomas”[All Fields] OR “Squamous Cell Carcinoma”[All Fields] OR “carcinoma squamous”[All Fields] OR “carcinomas squamous”[All Fields] OR “Squamous Carcinoma”[All Fields] OR “Squamous Carcinomas”[All Fields] OR “carcinoma epidermoid”[All Fields] OR “carcinomas epidermoid”[All Fields] OR “Epidermoid Carcinoma”[All Fields] OR “Epidermoid Carcinomas”[All Fields] OR (“carcinoma, squamous cell”[MeSH Terms] OR (“carcinoma”[All Fields] AND “squamous”[All Fields] AND “cell”[All Fields]) OR “Squamous Cell Carcinoma”[All Fields] OR (“carcinoma”[All Fields] AND “planocellular”[All Fields])) OR “carcinomas planocellular”[All Fields] OR “Planocellular Carcinoma”[All Fields] OR “Planocellular Carcinomas”[All Fields] OR “Squamous Cell Carcinoma of Head and Neck”[MeSH Terms] OR “Head And Neck Squamous Cell Carcinomas”[All Fields] OR “squamous cell carcinoma head and neck”[All Fields] OR “Squamous Cell Carcinoma of the Head and Neck”[All Fields] OR “Head and Neck Squamous Cell Carcinoma”[All Fields] OR “HNSCC”[All Fields] OR “carcinoma squamous cell of head and neck”[All Fields] OR “Squamous Cell Carcinoma of the Larynx”[All Fields] OR “Laryngeal Squamous Cell Carcinoma”[All Fields] OR “Squamous Cell Carcinoma of Larynx”[All Fields] OR “Squamous Cell Carcinoma of the Nasal Cavity”[All Fields] OR “Oral Tongue Squamous Cell Carcinoma”[All Fields] OR “Hypopharyngeal Squamous Cell Carcinoma”[All Fields] OR “Oral Squamous Cell Carcinoma”[All Fields] OR “Oral Cavity Squamous Cell Carcinoma”[All Fields] OR “Oral Squamous Cell Carcinomas”[All Fields] OR “Squamous Cell Carcinoma of the Mouth”[All Fields] OR “Oropharyngeal Squamous Cell Carcinoma”[All Fields]) AND (“Food”[MeSH Terms] OR “Foods”[All Fields] OR “diet, food, and nutrition”[MeSH Terms] OR “Diet”[MeSH Terms] OR “Diets”[All Fields] OR “Eating”[MeSH Terms] OR “Food Intake”[All Fields] OR “intake food”[All Fields] OR “Macronutrient Intake”[All Fields] OR “intake macronutrient”[All Fields] OR “Macronutrient Intakes”[All Fields] OR “Dietary Intake”[All Fields] OR “Dietary Intakes”[All Fields] OR “intake dietary”[All Fields] OR “Micronutrient Intake”[All Fields] OR “intake micronutrient”[All Fields] OR “Micronutrient Intakes”[All Fields] OR “Ingestion”[All Fields] OR “Feed Intake”[All Fields] OR “Feed Intakes”[All Fields] OR “intake feed”[All Fields] OR “Nutrient Intake”[All Fields] OR “intake nutrient”[All Fields] OR “Nutrient Intakes”[All Fields] OR “Nutritional Intake”[All Fields] OR “intake nutritional”[All Fields] OR “Nutritional Intakes”[All Fields]) AND (“cohort studies”[MeSH Terms] OR “case-control studies”[MeSH Terms] OR “comparative study”[Publication Type] OR “risk factors”[MeSH Terms] OR “cohort”[Text Word] OR “compared”[Text Word] OR “groups”[Text Word] OR “case control”[Text Word] OR “multivariate”[Text Word])
LILACS (by BVS)<br>92	#1 “Carcinoma de Células Escamosas” OR “Carcinoma, Squamous Cell” OR “Carcinome épidermoïde” OR “Carcinoma de Células Escamosas de Cabeça e Pescoço” OR “Squamous Cell Carcinoma of Head and Neck” OR “Carcinoma de Células Escamosas de Cabeza y Cuello” OR “Carcinome épidermoïde de la tête et du cou”<br>#2 “Alimentos” OR “Food” OR “Alimentos” OR “Aliments” OR “Dieta” OR “Diet” OR “Régime alimentaire” OR “Ingestão de Alimentos” OR “Eating” OR “Ingestión de Alimentos” OR “Consommation alimentaire”
Embase<br>3225	‘clinical article’/exp OR ‘controlled study’/exp OR ‘major clinical study’/exp OR ‘prospective study’/exp OR ‘cohort analysis’/exp OR ‘cohort’:ti,ab OR ‘compared’:ti,ab OR ‘groups’:ti,ab OR ‘case control’:ti,ab OR ‘multivariate’:ti,ab OR ‘head and neck squamous cell carcinoma’:ab,ti AND [embase]/lim OR (‘head and neck squamous cell carcinoma’/exp OR ‘head and neck squamous cell carcinoma’/syn) AND [embase]/lim OR ‘mouth squamous cell carcinoma’:ab,ti AND [embase]/lim OR (‘mouth squamous cell carcinoma’/exp OR ‘mouth squamous cell carcinoma’/syn) AND [embase]/lim OR (food:ab,ti OR ‘nutrition’:ab,ti OR diet:ab,ti OR ‘dietary intake’:ab,ti) AND [embase]/lim OR (‘dietary intake’/exp OR ‘dietary intake’/syn) AND [embase]/lim OR (‘diet’/exp OR

	'diet'/syn) AND [embase]/lim OR ('nutrition'/exp OR 'nutrition'/syn) AND [embase]/lim OR ('food'/exp OR 'food'/syn) AND [embase]/lim
Web of Science 1302	("Carcinoma de Células Escamosas" OR "Carcinoma, Squamous Cell" OR "Carcinome épidermoïde" OR "Carcinoma de Células Escamosas de Cabeça e Pescoço" OR "Squamous Cell Carcinoma of Head and Neck" OR "Carcinoma de Células Escamosas de Cabeza y Cuello" OR "Carcinome épidermoïde de la tête et du cou") AND ("Alimentos" OR "Food" OR "Alimentos" OR "Aliments" OR "Dieta" OR "Diet" OR "Régime alimentaire" OR "Ingestão de Alimentos" OR "Eating" OR "Ingestión de Alimentos" OR "Consommation alimentaire") AND (db:("WPRIM" OR "LILACS" OR "IBECS" OR "BBO" OR "BINACIS" OR "CUMED" OR "BRISA"))

Table S2. Full-text excluded articles and reasons.

Author (year)	Reference	Exclusion Motif
Abe et al., 2021	[1]	2
Amin, Biswas, Ahmed (2015)	[2]	3
Amtha et al., 2009*	[3]	8
Anwar et al., 2020	[4]	3
Ayub et al., 2015	[5]	2
Bansal and Gupta (2022)	[6]	4
Bell et al., 2021	[7]	1
Boeing et al., 2006	[8]	1
Bravi et al., 2013	[9]	1
Brunnoto et al., 2019*	[10]	8
Casto et al., 2013	[11]	4
Chatelain et al., 2011	[12]	4
Das, Gheena, Kumar (2020)	[13]	2
Don et al., 2022*	[14]	8
Dwyer et al., 1991	[15]	7
Harada et al., 2019	[16]	2
Hassabou and Farag (2020)	[17]	4
Huang et al., 2017	[18]	5
Jagtap and Tele (2020)	[19]	6
Kansara et al., 2019	[20]	2
Kaokaen et al., 2020	[21]	4
Kapila et al., 2006	[22]	4
Kato et al., 2008	[23]	4
Keshani, et al.,2023*	[24]	8
Kune et al., 1993	[25]	1
Lawal et al., 2011	[26]	2
Liao et al., 2007	[27]	2
Liu et al., 2006	[28]	3
Llewellyn, et al.,2004*	[29]	8
Nagle et al., 2015	[30]	1
Petti S. (2009)	[31]	7
Ren et al., 2010*	[32]	8
Rodríguez-Molinero et al., 2021	[33]	4
Shirataki et al., 2000	[3[4]	4
Stefani et al., 2005*	[35]	8
Stefani et al., 2000	[36]	1
Stucken,Weissman and Spiegel (2010)	[37]	7
Sundermann et al., 2018	[38]	2

Trachootham et al., 2017	[39]	4
Turati et al., 2011	[40]	4
Vieytes et al., 2023*	[41]	8
Winn DM. (1995)	[42]	1
Wyk et al., 1993	[43]	3
Yan et al., 2016	[44]	5
Yang et al., 2021	[45]	4

Legend—Exclusion criteria: 1—Histopathological diagnosis incompatible with OSCC (n = 7); 2—No diet association (n = 8); 3—Use of herbs (n = 4); 4—Type study design (n = 12); 5—Language (n = 2); 6—Age (n = 1); 7—No access, authors contacted without reply (n = 3); 8 - Excluded from quantitative analysis due to lack of quantitative food consumption data (n = 8)*.

References

1. Wyk, van; Stander, I.; Padayachee, A.; Grobler-Rabie, A.F. The Areca Nut Chewing Habit and Oral Squamous Cell Carcinoma in South African Indians. A Retrospective Study. *PubMed* **1993**, *83*, 425–429.
2. Sundermann, B.V.; Uhlmann, L.; Hoffmann, J.; Freier, K.; Thiele, O.C. The Localization and Risk Factors of Squamous Cell Carcinoma in the Oral Cavity: A Retrospective Study of 1501 Cases. *Journal of Cranio-Maxillofacial Surgery* **2018**, *46*, 177–182, doi:https://doi.org/10.1016/j.jcms.2017.10.019.
3. Llewellyn, C.D.; Linklater, K.; Bell, J.; Johnson, N.W.; Warnakulasuriya, S. An Analysis of Risk Factors for Oral Cancer in Young People: A Case-Control Study. *Oral Oncology* **2004**, *40*, 304–313, doi:https://doi.org/10.1016/j.oraloncology.2003.08.015.
4. Lawal , A.; Kolude , B.; Adeyemi , B.; Lawoyin, J.; Akang, E. Social Profile and Habits of Oral Cancer Patients in Ibadan. *PubMed* **2011**, *40*, 247–251.
5. Kune, G.A.; Kune, S.; Field, B.; Watson, L.F.; Cleland, H.; Merenstein, D.; Vitetta, L. Oral and Pharyngeal Cancer, Diet, Smoking, Alcohol, and Serum Vitamin a and Β-Carotene Levels: A Case-Control Study in Men. *Nutrition and Cancer* **1993**, *20*, 61–70, doi:https://doi.org/10.1080/01635589309514271.
6. Nagle, C.M.; Wilson, L.F.; Hughes, M.C.B.; Ibiebele, T.I.; Miura, K.; Bain, C.J.; Whiteman, D.C.; Webb, P.M. Cancers in Australia in 2010 Attributable to Inadequate Consumption of Fruit, Non-Starchy Vegetables and Dietary Fibre. *Australian and New Zealand Journal of Public Health* **2015**, *39*, 422–428, doi:https://doi.org/10.1111/1753-6405.12449.
7. Petti, S. Lifestyle Risk Factors for Oral Cancer. *Oral Oncology* **2009**, *45*, 340–350, doi:https://doi.org/10.1016/j.oraloncology.2008.05.018.
8. Stucken, E.; Weissman, J.; Spiegel, J.H. Oral Cavity Risk Factors: Experts' Opinions and Literature Support. *PubMed* **2010**, *39*, 76–89.
9. Winn, D.M. Diet and Nutrition in the Etiology of Oral Cancer. *The American Journal of Clinical Nutrition* **1995**, *61*, 437S445S, doi:https://doi.org/10.1093/ajcn/61.2.437s.
10. Amtha, R.; Zain, R.; Razak, I.A.; Basuki, B.; Roeslan, B.O.; Gautama, W.; Purwanto, D.J. Dietary Patterns and Risk of Oral Cancer: A Factor Analysis Study of a Population in Jakarta, Indonesia. *Oral Oncology* **2009**, *45*, e49–e53, doi:https://doi.org/10.1016/j.oraloncology.2009.01.007.
11. Stefani, E.D.; Boffetta, P.; Ronco, A.L.; Correa, P.; Oreggia, F.; Deneo-Pellegrini, H.; Mendilaharsu, M.; Leiva, J.C. Dietary Patterns and Risk of Cancer of the Oral Cavity and Pharynx in Uruguay. *Nutrition and Cancer* **2005**, *51*, 132–139, doi:https://doi.org/10.1207/s15327914nc5102_2.
12. Don, J.; Secchi, D.G.; María Fernanda Galíndez; Aballay, L.R.; María Eugenia Pasqualini; Brunotto, M. The Association among TP53 Rs1042522, Pri-MiR 34b/c Rs4938723 Polymorphisms and Daily Dietary Fatty Acids in Patients with Premalignant and Malignant Oral Lesions. *Human Gene* **2022**, *34*, 201082–201082, doi:https://doi.org/10.1016/j.humgen.2022.201082.
13. Keshani, F.; Razavi, S.; Askari, G.; Zahiri, Z.; Heidari, Z. A Comparative Analysis of Dominant Dietary Patterns in Patients with and without Oral Squamous Cell Carcinoma. *Advanced Biomedical Research* **2023**, *12*, 4–4, doi:https://doi.org/10.4103/abr.abr_120_21.
14. Brunotto, M.; Secchi, D.G.; Aballay, L.R.; Shivappa, N.; Hebert, J.R.; Galíndez Costa, M.F. The Inflammatory Potential of Argentinian Diet and Oral Squamous Cell Carcinoma. *Nutrición Hospitalaria* **2019**, doi:https://doi.org/10.20960/nh.02613.
15. Vieytes, C.A.M.; Rozek, L.S.; Wolf, G.T.; Arthur, A.E. Associations between Diet Quality and Proinflammatory Cytokines in Newly Diagnosed Head and Neck Cancer Survivors. *Current Developments in Nutrition* **2023**, *7*, 102015–102015, doi:https://doi.org/10.1016/j.cdnut.2023.102015.
16. Ren, J.S.; Freedman, N.D.; Kamangar, F.; Dawsey, S.M.; Hollenbeck, A.R.; Schatzkin, A.; Abnet, C.C. Tea, Coffee, Carbonated Soft Drinks and Upper Gastrointestinal Tract Cancer Risk in a Large United States Prospective Cohort Study. *European Journal of Cancer* **2010**, *46*, 1873–1881, doi:https://doi.org/10.1016/j.ejca.2010.03.025.
17. Harada, K.; Fujiwara, R.; Hisano, T.; Takenawa, T.; Mishima, K. SUN-PO110: Basic Investigation on the Mechanisms of Action of Elemental Diet Elental® in Oral Cancer Treatment. *Clinical Nutrition* **2019**, *38*, S100, doi:https://doi.org/10.1016/s0261-5614(19)32744-x.
18. Dwyer, J.T.; Efstathion, A.; Palmer, C.; Papas, A. Nutritional Support in Treatment of Oral Carcinomas. *Nutrition Reviews* **1991**, *49*, 332–337.

19. Abe, A.; Hayashi, H.; Ishihama, T.; Furuta, H. Prognostic Impact of the Prognostic Nutritional Index in Cases of Resected Oral Squamous Cell Carcinoma: A Retrospective Study. *BMC Oral Health* **2021**, *21*, doi:https://doi.org/10.1186/s12903-021-01394-6.
20. Amin, N.; Biswas, S.L.; Ahmed, M. Study of Squamous Cell Carcinoma in a Tertiary Level Hospital in Bangladesh. *International Journal of Oral and Maxillofacial Surgery* **2015**, *44*, e27, doi:https://doi.org/10.1016/j.ijom.2015.08.444.
21. Das, A.; Gheena, N.S.; Kumar , J.R. Age and Gender Predilection of Habits and Oral Cancer among an Outpatient Population Visiting a Dental Hospital. *International Journal of Research in Pharmaceutical Sciences* **2020**, *11*, 1850–1854, doi:https://doi.org/10.26452/ijrps.v11ispl3.3547.
22. Ayub, M.; fayyaz, M.; arshad, S.; khan, B. riaz; bano, U.; khalil, F. Oral Cancer Prevalence & Finding of Alarming Consequences at Oncology Ward of Public Health Care Sector. *International Research Journal of Pharmacy* **2015**, *6*, 623–626, doi:https://doi.org/10.7897/2230-8407.069121.
23. Bansal, M.; Gupta, T.K. Dietary Risk Factors in Upper Aero-Digestive Tract Cancers. *Indian Journal of Otolaryngology and Head and Neck Surgery* **2022**, *74*, 6356–6361, doi:https://doi.org/10.1007/s12070-022-03093-2.
24. Bell, E.B.; Reis, I.M.; Cohen, E.R.; Almuhaimid, T.; Smith, D.H.; Alotaibi, F.; Gordon, C.; Gomez-Fernandez, C.; Goodwin, W.J.; Franzmann, E.J. Green Salad Intake Is Associated with Improved Oral Cancer Survival and Lower Soluble CD44 Levels. *Nutrients* **2021**, *13*, 372–372, doi:https://doi.org/10.3390/nu13020372.
25. Boeing, H.; Dietrich, T.; Hoffmann, K.; Pischon, T.; Ferrari, P.; Lahmann, P.H.; Boutron-Ruault, M.C.; Clavel-Chapelon, F.; Allen, N.; Key, T.; et al. Intake of Fruits and Vegetables and Risk of Cancer of the Upper Aero-Digestive Tract: The Prospective EPIC-Study. *Cancer Causes & Control* **2006**, *17*, 957–969, doi:https://doi.org/10.1007/s10552-006-0036-4.
26. Bravi, F.; Bosetti, C.; Filomeno, M.; Levi, F.; Garavello, W.; Galimberti, S.; Negri, E.; La Vecchia, C. Foods, Nutrients and the Risk of Oral and Pharyngeal Cancer. *British Journal of Cancer* **2013**, *109*, 2904–2910, doi:https://doi.org/10.1038/bjc.2013.667.
27. Casto, B.C.; Knobloch, T.J.; Galioto, R.L.; Yu, Z.; Accurso, B.T.; Warner, B.M. Chemoprevention of Oral Cancer by Lyophilized Strawberries. *Anticancer Research* **2013**, *33*, 4757–4766.
28. Huang, J.; Qiu, Y.; Cai, L.; Liu, F.; Chen, F.; Yan, L.; Wu, J.; Bao, X.; Liu, F.; Zheng, X.; et al. [Pickled Food, Fish, Seafood Intakes and Oral Squamous Cell Carcinoma: A Case-Control Study]. *PubMed* **2017**, *51*, 680–685, doi:https://doi.org/10.3760/cma.j.issn.0253-9624.2017.08.005.
29. Chatelain, K.; Phippen, S.; McCabe, J.; Teeters, C.A.; O'Malley, S.; Kingsley, K. Cranberry and Grape Seed Extracts Inhibit the Proliferative Phenotype of Oral Squamous Cell Carcinomas. *Evidence-Based Complementary and Alternative Medicine* **2011**, *2011*, 1–12, doi:https://doi.org/10.1093/ecam/nen047.
30. Yan, L.; Chen, F.; Liu, D.; Huang, J.; Liu, F.; Wu, J.; Liu, F.; Ye, J.; Qiu, Y.; Lin, L.; et al. [Tea, Coffee Intakes and Risk of Oral Squamous Cell Carcinoma: A Case-Control Study]. *PubMed* **2016**, *37*, 1531–1535, doi:https://doi.org/10.3760/cma.j.issn.0254-6450.2016.11.019.
31. Kaokaen, P.; Jaiboonma, A.; Chaicharoenaudomrung, N.; Kunhorm, P.; Janebodin, K.; Noisa, P.; Jitprasertwong, P. Cordycepin-Loaded Nanoparticles from Cassava Starch Promote the Proliferation of Submandibular Gland Cells and Inhibit the Growth of Oral Squamous Carcinoma Cells. *Nutrition and Cancer* **2020**, *73*, 2014–2029, doi:https://doi.org/10.1080/01635581.2020.1819350.
32. Hassabou, N.F.; Farag, A.F. Anticancer Effects Induced by Artichoke Extract in Oral Squamous Carcinoma Cell Lines. *Journal of the Egyptian National Cancer Institute* **2020**, *32*, doi:https://doi.org/10.1186/s43046-020-00026-4.
33. Jagtap, S.V.; Tele, J. Clinicohistopathological Profile of Malignant and Pre-Malignant Lesions of Oral Cavity. *International Journal of Research in Pharmaceutical Sciences* **2020**, *11*, 5729–5734.
34. Kansara, S.; Wang, T.; Koochakzadeh, S.; Liou, N.E.; Graboyes, E.M.; Skoner, J.M.; Hornig, J.D.; Sandulache, V.C.; Day, T.A.; Huang, A.T. Prognostic Factors Associated with Achieving Total Oral Diet Following Osteocutaneous Microvascular Free Tissue Transfer Reconstruction of the Oral Cavity. *Oral Oncology* **2019**, *98*, 1–7, doi:https://doi.org/10.1016/j.oraloncology.2019.09.006.
35. Kato, K.; Long, N.K.; Makita, H.; Toida, M.; Yamashita, T.; Hatakeyama, D.; Hara, A.; Mori, H.; Shibata, T. Effects of Green Tea Polyphenol on Methylation Status of RECK Gene and Cancer Cell Invasion in Oral Squamous Cell Carcinoma Cells. *British Journal of Cancer* **2008**, *99*, 647–654, doi:https://doi.org/10.1038/sj.bjc.6604521.
36. Kapila, A.R.; Rawal, Y.; Renner, R.J.; Schwartz, S.J.; Tian, Q.; Larsen, P.E.; Mallery, S.R. Suppression of the Tumorigenic Phenotype in Human Oral Squamous Cell Carcinoma Cells by an Ethanol Extract Derived from Freeze-Dried Black Raspberries. *Nutrition and Cancer* **2006**, *54*, 58–68, doi:https://doi.org/10.1207/s15327914nc5401_7.
37. Liao, C.-T.; Chang, J.T.-C.; Wang, H.-M.; Ng, S.-H.; Hsueh, C.; Lee, L.-Y.; Lin, C.-H.; Chen, I-How.; Huang, S.-F.; Cheng, A.-J.; et al. Analysis of Risk Factors of Predictive Local Tumor Control in Oral Cavity Cancer. *Annals of Surgical Oncology* **2007**, *15*, 915–922, doi:https://doi.org/10.1245/s10434-007-9761-5.
38. Liu, S.-A.; Tsai, W.-C.; Wong, Y.-K.; Lin, J.-C.; Poon, C.-K.; Chao, S.-Y.; Hsiao, Y.-L.; Chan, M.-Y.; Cheng, C.-S.; Wang, C.-C.; et al. Nutritional Factors and Survival of Patients with Oral Cancer. *Head & Neck* **2006**, *28*, 998–1007, doi:https://doi.org/10.1002/hed.20461.
39. Rodríguez-Molinero, J.; Migueláñez-Medrán, B. del C.; Puente-Gutiérrez, C.; Delgado-Somolinos, E.; Martín Carreras-Presas, C.; Fernández-Farhall, J.; López-Sánchez, A.F. Association between Oral Cancer and Diet: An Update. *Nutrients* **2021**, *13*, 1299, doi:https://doi.org/10.3390/nu13041299.
40. Trachootham, D.; Chingsuwanrote, P.; Yoosadiang, P.; Mekkriangkrai, D.; Ratchawong, T.; Buraphacheep, N.; Kijanukul, S.; Saekhow, S.; Pongpitchayadej, O.; Vongvachvasin, K.; et al. Partial Substitution of Glucose with Xylitol Suppressed the Glycolysis and Selectively Inhibited the Proliferation of Oral Cancer Cells. *Nutrition and Cancer* **2017**, *69*, 862–872, doi:https://doi.org/10.1080/01635581.2017.1339097.

41. Turati, F.; Galeone, C.; La Vecchia, C.; Garavello, W.; Tavani, A. Coffee and Cancers of the Upper Digestive and Respiratory Tracts: Meta-Analyses of Observational Studies. *Annals of Oncology* **2011**, *22*, 536–544, doi:https://doi.org/10.1093/annonc/mdq603.
42. Yang, M.; Luo, Q.; Chen, X.; Chen, F. Bitter Melon Derived Extracellular Vesicles Enhance the Therapeutic Effects and Reduce the Drug Resistance of 5-Fluorouracil on Oral Squamous Cell Carcinoma. *Journal of Nanobiotechnology* **2021**, *19*, doi:https://doi.org/10.1186/s12951-021-00995-1.
43. Anwar, N.; Pervez, S.; Chundriger, Q.; Awan, S.; Moatter, T.; Ali, T.S. Oral Cancer: Clinicopathological Features and Associated Risk Factors in a High Risk Population Presenting to a Major Tertiary Care Center in Pakistan. *PLOS ONE* **2020**, *15*, e0236359, doi:https://doi.org/10.1371/journal.pone.0236359.
44. Stefani, E.D.; Oreggia, F.; Boffetta, P.; Deneo-Pellegrini, H.; Ronco, A.; Mendilaharsu, M. Tomatoes, Tomato-Rich Foods, Lycopene and Cancer of the Upper Aerodigestive Tract: A Case-Control in Uruguay. *Oral Oncology* **2000**, *36*, 47–53, doi:https://doi.org/10.1016/S1368-8375(99)00050-0.
45. Shirataki, Y.; Kawase, M.; Saito, S.; Kurihara, T.; Tanaka, W.; Satoh, K.; Sakagami, H.; Motohashi, N. Selective Cytotoxic Activity of Grape Peel and Seed Extracts against Oral Tumor Cell Lines. *Anticancer Research* **2000**, *20*, 423–426.
